# Handgrip strength, dynapenia, and mental health in older Koreans

**DOI:** 10.1038/s41598-020-60835-4

**Published:** 2020-03-04

**Authors:** Hye-Mi Noh, Yong Soon Park

**Affiliations:** 1 Department of Family Medicine, Hallym University Sacred Heart Hospital, Anyang, Gyeonggi-do Republic of Korea; 2 Department of Family Medicine, Chuncheon Sacred Heart Hospital, Hallym University College of Medicine, Chuncheon, Republic of Korea; 3 Present Address: Department of Family Medicine, Chuncheon Sacred Heart Hospital, Hallym University College of Medicine, 77 Sakju-ro, Chuncheon, Gangwon-do 24253 Republic of Korea

**Keywords:** Diagnostic markers, Risk factors

## Abstract

This study examined associations between muscle strength and mental health among 2,652 elderly Koreans who participated in the 2015 and 2017 Korea National Health and Nutrition Examination Survey. We measured absolute handgrip strength and calculated handgrip strength relative to body mass index. Dynapenia criteria followed that of the Asian Working Group for Sarcopenia. Questionnaires were used to assess mental health indicators including suicidal ideation, depressed mood, and stress status. Among participants, 18.6%, 14.9%, and 6.7% reported stress, depressed mood, and suicidal ideation, respectively. Dynapenia prevalence was 25.1%. Adjusted odds ratios of stress, depressed mood, and suicidal ideation for men with dynapenia were 2.15, 2.30, and 2.11, respectively. Significant associations were absent among women. For men, handgrip strength and relative handgrip strength were inversely associated with risk of stress, depressed mood, and suicidal ideation. For women, handgrip strength and relative handgrip strength were inversely associated with the risk of stress, but not of depressed mood and suicidal ideation. In conclusion, all muscle-strength indices were associated with mental health risks among older men. In older women, low handgrip strength and relative handgrip strength were associated with increased stress. Muscle strength could be a clinical marker of poor mental health in older adults.

## Introduction

Korea has officially become an aged society. The number of people aged 65 years and older increased to 7.38 million in 2018, accounting for 14.3% of the total population^1^. This status emerged only 18 years after Korea became an aging society, when those aged 65 years and older accounted for over 7% of the population^1^. Unpreparedness for aging aggravates both physical and mental well-being of the elderly population. Older people experience increased weakness, loss of physical function, and greater risk of disease^2^. These physical changes lead to reduced roles in the home, workplace, and society, generating social and economic burdens for the elderly^3^. As a result, older individuals are at greater risk of mental health problems such as stress, depression, and suicide^4,5^. Older adults in Korea are among the most vulnerable to suicide, with a rate of about 72 per 100,000 people during 2010. This ratio is nearly five times higher than that in 1990 and about 3.5 times the mean suicide rate in countries of the Organization for Economic Cooperation Development^6^.

Sarcopenia is a decrease in skeletal muscle mass and strength that develops because of aging. The resultant physical frailty is associated with loss of independence, as well as increased risks of morbidity and mortality^7^. Recently, several studies showed that muscle strength is a better predictor of disability than muscle mass, while dynapenia (age-related loss of muscle strength) is independently associated with physical disability and mortality^8^. Currently, handgrip strength (HGS) is the only assessment technique recommended for measuring muscle strength and muscle function^9^. Being relatively simple, non-invasive, and inexpensive, the test is widely used to measure strength in older adults^10^. Low HGS predisposes individuals to poorer physical function and increased risk for a number of diseases^11^ while predicting future all-cause mortality^12^. Low HGS is closely related to various adverse health outcomes in Korean older adults^13,14^. Given its predictive validity and simplicity, HGS could be a useful health-screening tool for older patients in primary care.

Low HGS might cause poor mental health. Several studies have reported that low HGS was associated with an increased risk of depressive symptoms among older adults^15–18^. However, only one study has investigated the association between HGS and suicidal ideation, and it reported that low relative HGS was associated with suicidal ideation^19^. In addition, previous studies have used different muscle strength indices (dynapenia, absolute HGS, and relative HGS), which could confound each one’s relationship with mental health.

Therefore, the current study aimed to assess the relationship between HGS and indicators of poor mental health, namely perceived stress, depressed mood, and suicidal ideation. Hence, we investigated whether three muscle strength indices (dynapenia, absolute HGS, and relative HGS) have a consistent association with mental health indicators.

## Methods

### Study population

**Figure 1:**
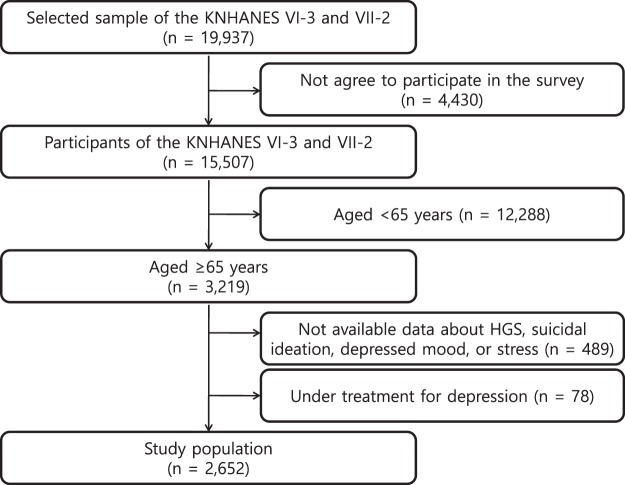
Flowchart showing selection of the study population.

Data were obtained from the 2015 and 2017 KNHANES (VI-3 and VII-2), conducted periodically since 1998 by the Korea Center for Disease Control and Prevention (KCDC) to assess non-institutionalised civilian health and nutritional status. Data from the 2016 KNHANES (VII-1) were excluded because it did not include suicidal ideation and depressed mood, variables that were investigated every alternative year. The KNHANES VI-3 and VII-2 are cross-sectional and nationally representative. Using a complex, stratified, multistage probability-cluster sampling design, the KCDC selected 19,937 individuals from 8,256 households for possible participation in the two surveys. Of those selected, 15,507 agreed to participate, yielding a response rate of 77.8%. The current study examined participants who met the inclusion criterion of being aged 65 years or older (*n* = 3,219). Participants were excluded if they had incomplete data for HGS (*n* = 398), suicidal ideation (*n* = 250), depressed mood (*n* = 250), or stress (*n* = 254). Participants under treatment for depression (*n* = 78) were also excluded. A total of 2,652 participants were analysed (Fig. 1). Ethical approval from an institutional review board was not required because the survey data are publicly available.

### KNHANES data collection and measurements

Data were collected through standardised health examinations in specially equipped mobile examination centres. The health survey was conducted in the following order: enrolment, receipt of written informed consent, anthropometric measurements, handgrip measurements, and questionnaire completion. The latter collected data on age, sex, sociodemographic factors, economic status, medical history, lifestyle-related risk factors, and mental health indicators.

Age was based on actual date of birth. The raw KNHANES data used age as a top-coding category to protect personal information. For analyses in the present study, we divided age into four categories at intervals of 5 years. Unmarried, separated, widowed, and divorced participants were assigned a ‘no spouse’ status. Participants were also divided into employed and unemployed. Employed individuals included those who worked over one hour for income, or over 18 hours as unpaid family workers, for one week (including temporary leave of absence). Monthly household income was divided into quartiles and grouped into lowest, mid-low/mid-high, and highest.

A self-report questionnaire was used to collect data regarding medical history for 14 clinically diagnosed chronic diseases (cancer, ischemic heart disease, stroke, hypertension, dyslipidaemia, diabetes mellitus, osteoarthritis, rheumatoid arthritis, osteoporosis, asthma, chronic obstructive pulmonary disease, thyroid disease, renal failure, and liver cirrhosis). Multimorbidity was defined as any co-occurrence of medical conditions within an individual^20^.

Smoking status was categorised into current smokers (smoked ≥100 cigarettes throughout lifetime and currently smoke), ex-smokers (smoked ≥100 cigarettes throughout lifetime but currently non-smoking), and never smokers (smoked <100 cigarettes throughout lifetime and currently non-smoking). Drinkers were participants who consumed alcohol at least once every month over the past year. Physically active participants were those who performed medium-intensity exercise for least 150 minutes per week, 75 minutes of high-intensity exercise per week, or a combination of medium- and high-intensity exercise (1 minute of high intensity = 2 minutes of medium intensity). Unintended weight loss was self-reported drops of 3 kg or more during the past year.

Anthropometric data were measured according to standardised guidelines. Height, body weight, and waist circumference (WC) were measured with participants wearing light clothing without shoes. To obtain WC (in tenths of a centimetre), participants were asked to stand with their feet 25–30 cm apart. The measurement was taken without compression of soft tissue along the horizontal line, equidistant between the inferior margin of the last rib and the iliac crest. Body mass index (BMI) was calculated by dividing body weight by height squared (kg/m^2^).

### Handgrip strength and dynapenia

Handgrip strength was measured with a digital grip strength dynamometer (TKK 5401; Takei Scientific Instruments Co., Ltd., Tokyo, Japan). Four well-trained staff members simultaneously measured HGS using the same device in each mobile screening vehicle. HGS measurement was performed before blood sampling or pulmonary function tests. Staff instructed participants to remove jewellery from their fingers or wrists and explained the measurement method and procedure in detail. Participants were instructed to hold the dynamometer while standing upright and keeping their arms at their sides. Participants gripped the instrument as tightly as they could for 3 seconds, three times per hand. A resting interval of at least 60 seconds was allowed between each measurement. The dominant hand’s highest HGS score was used for analysis. Relative HGS was calculated as HGS divided by BMI. According to the Asian Working Group for Sarcopenia (AWGS), dynapenia is defined as HGS <26 kg for men and <18 kg for women^21^.

### Mental health indicators

Suicidal ideation was assessed using the question: ‘Have you ever seriously considered suicide in the last year?’ Depressed mood was assessed using the question: ‘Have you ever felt sad or hopeless enough to cause disruptions to your daily life for more than two consecutive weeks in the past year?’ Participants could only answer yes or no, and the validity of this question was evaluated using previous studies^22^. To assess perceived stress, participants were asked: ‘How much stress do you usually feel during your daily life?’ This single item measure of perceived stress has been previously validated^23^. Responses were recorded using a four-point scale: almost none, some, high, and very high. Participants were considered stressed if they reported high or very high stress.

### Statistical analysis

Estimates were weighted based on sampling rate, response rate, and age and sex proportions of the reference population. Analyses were adjusted for the complex sample design of the survey. Continuous data are presented as means ± SE, while categorical data are presented as weighted proportion (SE). General characteristics were compared between men and women using the Student’s *t*-test for continuous data and chi-square test for categorical data. Analyses of relationships between mental health indicators and independent variables (e.g., demographic characteristics) were performed separately for each sex. Chi-square tests were used to identify relationships between mental health indicators and independent variables. Age-adjusted logistic regressions were used to examine associations between muscle strength indices and mental health indicators. The confounding effects of potential risk factors were controlled for with multiple logistic regression. All tests were two-sided, and significance was set at P < 0.05. Analyses were performed in SPSS version 23.0 (IBM Co., Armonk, NY, USA).

### Ethical approval and informed consent

Participants submitted a written informed consent form before participating in the surveys. The 2014–2015 KNHANES was approved by the Institutional Review Board of the Korea Center for Disease Control and Prevention (2013-12EXP-03-5C). Since 2014, the KNHANES has been exempted from review about research ethics based on the Bioethics and Safety Act. All procedures performed in studies involving human participants were in accordance with the ethical standards of the institutional and/or national research committee.

## Results

### General characteristics of participants by sex

Of the 2,652 participants, 1,443 participants (55.4%) were women; 18.6, 14.9, and 6.7% of the study population reported stress, depressed mood, and suicidal ideation, respectively. Dynapenia prevalence was 25.1%. Women were significantly older than men and more likely than men to have no spouse, less education, lower economic status, multimorbidity, physical inactivity, dynapenia, depressed mood, and higher stress (all P < 0.001; Table 1). In contrast, men were more likely than women to report smoking (P < 0.001), alcohol consumption (P < 0.001), and unintended weight loss (P = 0.037). Men and women did not differ in terms suicidal ideation, with 6.2% of men and 7.1% of women having suicidal thoughts.

**Table 1:** General characteristics of participants by sex.

Characteristics	Men (*n* = 1,209)			Women (*n* = 1,443)			P
Age (years)							<0.001
≥80	174	14.5	(1.1)	246	17.6	(1.4)	
75−79	274	21.2	(1.2)	338	27.3	(1.4)	
70−74	334	27.3	(1.4)	375	23.7	(1.3)	
65−69	427	37.0	(1.6)	484	31.4	(1.3)	
Spouse: No	144	11.2	(1.0)	738	52.5	(1.7)	<0.001
Education: ≤Elementary school	462	40.7	(1.9)	987	73.6	(1.4)	<0.001
Employment: No	667	59.6	(2.0)	980	74.1	(1.5)	<0.001
Household income							<0.001
Lowest quartile	457	36.8	(1.7)	724	51.3	(1.8)	
Middle	604	51.1	(1.7)	585	40.1	(1.6)	
Highest quartile	143	12.1	(1.3)	127	8.7	(1.1)	
Residence: Rural area	332	25.1	(3.2)	421	26.6	(2.9)	0.434
Multimorbidity							<0.001
≥3	228	17.8	(1.3)	606	41.2	(1.5)	
1–2	676	55.7	(1.7)	659	45.8	(1.5)	
0	305	26.5	(1.4)	178	13.0	(1.1)	
Smoking							<0.001
Current smoker	232	18.8	(1.4)	27	2.0	(0.5)	
Ex-smoker	732	61.9	(1.7)	52	3.5	(0.5)	
Never smoker	245	19.4	(1.3)	1,363	94.5	(0.7)	
Alcohol drink: ≥1/month	708	58.9	(1.6)	270	18.5	(1.0)	<0.001
Physical activity: No	713	62.4	(1.7)	989	72.8	(1.5)	<0.001
Unintended weight loss: ≥3 kg/year	158	13.5	(1.2)	143	10.3	(1.0)	0.037
Body mass index, kg/m^2^		23.6	± 0.1		24.5	± 0.1	<0.001
Waist circumference, cm		86.3	± 0.3		84.4	± 0.3	<0.001
Dynapenia (<26/18 kg)	168	13.5	(1.1)	496	34.5	(1.7)	<0.001
Handgrip strength		33.2	± 0.2		20.1	± 0.2	<0.001
Relative handgrip strength		1.42	± 0.01		0.83	± 0.01	<0.001
Stress	160	12.6	(1.1)	329	23.4	(1.3)	<0.001
Depressed mood	137	11.0	(1.0)	262	18.1	(1.2)	<0.001
Suicidal ideation	76	6.2	(1.0)	101	7.1	(0.8)	0.411

Data are expressed as the estimated mean ± standard error or frequency, estimated percentage (standard error), as appropriate. P-values are derived from Student’s t-tests (means) or chi-square tests (proportions).

### Factors associated with stress, depressed mood, or suicidal ideation

Regardless of sex, participants with no spouse, less education, and low household income were more likely to report depressed mood (all P < 0.05; Tables 2 and 3). Moreover, lower household income was significantly associated with suicidal ideation for both sexes (P < 0.05). Older men with dynapenia were significantly more likely to report stress (P = 0.035), depressed mood (P < 0.001), and suicidal ideation (P < 0.001), but these associations were not present among older women with dynapenia.

**Table 2:** Factors associated with stress, depressed mood, or suicidal ideation in older Korean men.

Characteristics	Stress		P	Depressed mood		P	Suicidal ideation		P
Age, years			0.205			0.402			0.045
≥80	7.7	(1.9)		12.2	(2.7)		9.2	(2.7)	
75−79	14.0	(2.5)		13.7	(2.4)		8.8	(2.0)	
70−74	14.5	(2.5)		9.3	(1.6)		4.9	(1.2)	
65−69	12.4	(1.8)		10.2	(1.6)		4.5	(1.1)	
Spouse			0.154			0.001			<0.001
No	16.7	(3.4)		20.6	(3.7)		14.1	(3.7)	
Yes	12.1	(1.1)		9.8	(1.0)		5.2	(0.8)	
Education			0.047			0.001			0.001
≤Elementary school	14.7	(1.9)		14.7	(2.0)		9.8	(2.3)	
≥Middle school	10.3	(1.3)		7.8	(1.1)		3.6	(0.7)	
Employment			0.084			0.028			0.064
No	10.7	(1.4)		12.6	(1.5)		7.3	(1.3)	
Yes	14.3	(1.7)		7.7	(1.4)		4.4	(1.2)	
Household income			0.172			<0.001			0.025
Lowest quartile	15.4	(2.0)		15.1	(1.9)		9.9	(1.7)	
Middle	11.3	(1.4)		10.3	(1.5)		4.1	(1.0)	
Highest quartile	10.4	(3.1)		1.7	(1.0)		3.9	(2.7)	
Residence			0.934			0.213			0.112
Rural area	12.8	(2.1)		13.1	(2.1)		9.0	(2.6)	
Urban area	12.6	(1.3)		10.3	(1.1)		5.2	(0.9)	
Multimorbidity			0.416			0.178			0.730
≥3	14.7	(2.5)		14.6	(2.5)		6.8	(1.7)	
1–2	11.5	(1.2)		10.7	(1.4)		6.5	(1.5)	
0	13.5	(2.2)		9.2	(1.7)		5.1	(1.3)	
Smoking			0.021			0.062			0.111
Current smoker	15.5	(2.4)		15.4	(2.8)		8.3	(2.3)	
Ex-smoker	13.4	(1.5)		10.4	(1.2)		6.5	(1.4)	
Never smoker	7.3	(1.6)		8.5	(1.7)		3.0	(1.0)	
Drinks alcohol			0.405			0.385			0.587
≥1/month	13.4	(1.4)		10.3	(1.2)		6.5	(1.3)	
<1/month	11.6	(1.7)		12.0	(1.6)		5.7	(1.2)	
Physical activity			0.738			0.113			0.353
No	12.4	(1.4)		11.9	(1.4)		6.8	(1.4)	
Yes	11.7	(1.6)		8.4	(1.6)		5.0	(1.3)	
Unintended weight loss: ≥3 kg/y			0.886			0.954			0.195
Yes	12.3	(2.5)		11.1	(2.8)		8.3	(2.3)	
No	12.7	(1.2)		11.0	(1.0)		5.9	(0.9)	
Dynapenia: <26 kg			0.035			<0.001			<0.001
Yes	18.7	(3.5)		22.1	(4.0)		12.9	(3.0)	
No	11.7	(1.1)		9.3	(0.9)		5.1	(0.9)	

Data are expressed as estimated percentages (standard error). P-values are derived from chi-square tests for proportions.

**Table 3:** Factors associated with stress, depressed mood, or suicidal ideation in older Korean women.

Characteristics	Stress		P	Depressed mood		P	Suicidal ideation		P
Age, years			0.281			0.293			0.117
≥80	18.4	(2.6)		20.2	(2.8)		10.5	(2.2)	
75−79	22.7	(2.7)		15.5	(2.4)		4.8	(1.3)	
70−74	24.4	(2.8)		16.1	(2.2)		8.1	(1.9)	
65−69	26.1	(2.5)		20.7	(2.3)		6.4	(1.3)	
Spouse			0.121			0.012			0.649
No	21.4	(1.7)		21.2	(1.9)		7.4	(1.1)	
Yes	25.6	(2.0)		14.8	(1.5)		6.7	(1.1)	
Education			0.071			0.004			0.148
≤Elementary school	25.2	(1.7)		20.2	(1.6)		7.5	(1.0)	
≥Middle school	19.4	(2.5)		12.3	(1.9)		5.2	(1.1)	
Employment			0.031			0.156			0.480
No	21.8	(1.6)		17.0	(1.4)		7.3	(1.0)	
Yes	29.0	(3.0)		21.5	(2.8)		5.9	(1.5)	
Household income			0.298			<0.001			0.014
Lowest quartile	25.3	(1.8)		24.5	(1.9)		9.1	(1.3)	
Middle	22.7	(2.2)		12.5	(1.5)		5.6	(1.0)	
Highest quartile	18.1	(4.2)		8.4	(3.1)		2.8	(1.4)	
Residence			0.941			0.129			0.037
Rural area	23.2	(2.5)		21.4	(2.6)		9.8	(1.7)	
Urban area	23.5	(1.6)		16.9	(1.4)		6.1	(0.8)	
Multimorbidity			0.371			0.573			0.739
≥3	22.7	(1.9)		19.6	(1.8)		6.4	(1.1)	
1–2	25.0	(1.9)		17.0	(1.7)		7.4	(1.2)	
0	19.8	(3.4)		17.3	(3.1)		8.2	(2.4)	
Smoking			0.417			0.511			0.229
Current smoker	23.1	(8.3)		9.7	(5.1)		1.4	(1.5)	
Ex-smoker	32.0	(7.7)		17.8	(6.0)		10.3	(4.5)	
Never	23.0	(1.4)		18.3	(1.2)		7.0	(0.8)	
Drinks alcohol			0.288			0.480			0.685
≥1/month	26.5	(3.4)		19.9	(3.0)		7.8	(2.0)	
<1/month	22.7	(1.5)		17.7	(1.2)		6.9	(0.8)	
Physical activity			0.516			0.162			0.082
No	24.3	(1.7)		19.3	(1.5)		7.7	(1.0)	
Yes	22.3	(2.6)		15.3	(2.2)		4.6	(1.2)	
Unintended weight loss: ≥3 kg/y			0.178			0.151			0.079
Yes	29.0	(4.7)		23.5	(4.3)		11.3	(3.1)	
No	22.7	(1.4)		17.5	(1.2)		6.6	(0.8)	
Dynapenia: <18 kg			0.668			0.503			0.630
Yes	24.1	(2.1)		19.3	(2.1)		7.6	(1.3)	
No	23.0	(1.7)		17.5	(1.5)		6.8	(0.9)	

Data are expressed as estimated percentage (standard error). P-values are derived from chi-square tests for proportions.

### Muscle strength index associated with stress, depressed mood, or suicidal ideation

Mean HGS and relative HGS were lower in older men with depressed mood than in those without it. In addition, HGS and relative HGS were both lower in men with suicidal ideation than without it. Among women, no mental health indicator was associated with differences in HGS and relative HGS (Table 4).

**Table 4:** Muscle strength index associated with stress, depressed mood, or suicidal ideation among older people in Korea.

Men	Stress					Depressed mood					Suicidal ideation				
	(+)		(−)		P	(+)		(−)		P	(+)		(−)		P
Body mass index, kg/m^2^	23.6	±0.3	23.6	±0.1	0.843	23.6	±0.4	23.6	±0.1	0.945	23.2	±0.4	23.7	±0.1	0.270
Waist circumference, cm	86.4	±0.9	86.3	±0.3	0.933	86.2	±1.0	86.3	±0.3	0.917	86.1	±1.3	86.3	±0.3	0.866
Handgrip strength, kg	32.1	±0.7	33.4	±0.3	0.077	31.0	±0.7	33.5	±0.2	0.002	29.5	±0.9	33.5	±0.2	<0.001
Relative handgrip strength	1.38	±0.03	1.43	±0.01	0.152	1.32	±0.03	1.43	±0.01	<0.001	1.29	±0.04	1.43	±0.01	0.002
Women	(+)		(−)		P	(+)		(−)		P	(+)		(−)		P
Body mass index, kg/m^2^	24.6	±0.2	24.5	±0.1	0.883	24.8	±0.2	24.5	±0.1	0.122	25.1	±0.4	24.5	±0.1	0.115
Waist circumference, cm	84.1	±0.5	84.6	±0.3	0.387	84.8	±0.6	84.4	±0.3	0.507	84.5	±1.1	84.4	±0.3	0.922
Handgrip strength, kg	19.5	±0.3	20.2	±0.2	0.055	19.9	±0.4	20.1	±0.2	0.529	19.5	±0.6	20.1	±0.2	0.311
Relative handgrip strength	0.81	±0.01	0.84	±0.01	0.070	0.81	±0.02	0.83	±0.01	0.194	0.79	±0.03	0.83	±0.01	0.106

Data are expressed as estimated means ± standard error. P-values are derived from Student’s t-tests.

### Association between mental health indicators and muscle-strength indices

Multivariable-adjusted odds ratios (ORs) of stress, depressed mood, and suicidal ideation for older men with dynapenia were 2.15 (95% confidence interval [CI], 1.17–3.96), 2.30 (95% CI, 1.28–4.16), and 2.11 (95% CI, 1.05–4.26), respectively (Table 5). We did not identify significant associations between dynapenia and mental health indicators in older women. For men, a 1-kg decrease in HGS was associated with 1.05-, 1.04-, and 1.07-fold increases in risk of stress (95% CI, 1.01–1.08), depressed mood (95% CI, 1.01–1.08), and suicidal ideation (95% CI, 1.02–1.12), respectively. In addition, a 0.1-unit decrease in relative HGS was associated with 1.09-, 1.10-, and 1.12-fold increases in risk of stress (95% CI, 1.01–1.18), depressed mood (95% CI, 1.02–1.19), and suicidal ideation (95% CI, 1.01–1.26), respectively. For women, a 1-kg decrease in HGS and 0.1-unit decrease in relative HGS were respectively associated with 1.04- and 1.12-fold increases in risk of stress (95% CI, 1.01–1.08; 1.04–1.20), but not with risk of depressed mood or suicidal ideation.

**Table 5:** Logistic regression analyses to evaluate the association between mental health indicators and muscle-strength indices of older people in Korea.

		Men				Women			
		Model 1		Model 2		Model 1		Model 2	
		OR	(95% CI)	OR	(95% CI)	OR	(95% CI)	OR	(95% CI)
Stress									
	Dynapenia (<26/18 kg)	2.35	(1.37–4.04)	2.15	(1.17–3.96)	1.23	(0.91–1.67)	1.18	(0.86–1.61)
	Handgrip strength (per −1 kg)	1.04	(1.01–1.08)	1.05	(1.01–1.08)	1.05	(1.02–1.08)	1.04	(1.01–1.08)
	Relative handgrip strength (per −0.1)	1.08	(1.00–1.16)	1.09	(1.01–1.18)	1.10	(1.03–1.18)	1.12	(1.04–1.20)
Depressed mood									
	Dynapenia (<26/18 kg)	2.95	(1.70–5.11)	2.30	(1.28–4.16)	1.18	(0.81–1.72)	1.03	(0.69–1.53)
	Handgrip strength (per −1 kg)	1.06	(1.02–1.09)	1.04	(1.01–1.08)	1.02	(0.98–1.05)	1.01	(0.97–1.04)
	Relative handgrip strength (per −0.1)	1.12	(1.05–1.20)	1.10	(1.02–1.19)	1.07	(0.99–1.17)	1.04	(0.95–1.13)
Suicidal ideation									
	Dynapenia (<26/18 kg)	2.30	(1.32–4.04)	2.11	(1.05–4.26)	1.05	(0.64–1.74)	0.78	(0.45–1.35)
	Handgrip strength (per −1 kg)	1.08	(1.03–1.12)	1.07	(1.02–1.12)	1.02	(0.97–1.07)	0.99	(0.94–1.05)
	Relative handgrip strength (per −0.1)	1.13	(1.02–1.25)	1.12	(1.01–1.26)	1.09	(0.97–1.23)	1.04	(0.92–1.18)

Model 1: adjusted for age.

Model 2: adjusted for age, marital status, education level, employment, household income, residence, multimorbidity, smoking, alcohol intake, physical activity, body mass index (only dynapenia and handgrip strength), waist circumference, and unintended weight loss.

OR, odds ratio; CI, Confidence interval.

## Discussion

The present study investigated relationships between HGS and mental health in older adults. Analysis of our large, representative sample revealed that all muscle strength indices (dynapenia, HGS, and relative HGS) were associated with higher perceived stress, depressed mood, and suicidal ideation among older men. In older women, low HGS and relative HGS were associated with increases in perceived stress, but not in depressed mood or suicidal ideation. Our findings are in line with those of previous research linking dynapenia with increased risk of physical limitations, cardiovascular disease, and mortality^8^. AWGS have suggested that the HGS cut-off for diagnosing sarcopenia should be relatively lower in Asian populations than in Western populations due to ethnicity-based differences in body size, lifestyle, and culture^21^. Hence, in this study, we used the AWGS-recommended HGS values to determine dynapenia. We included relative HGS in addition to absolute HGS as the latter is related to body mass, which can confound attempts to examine health risks associated with HGS specifically^24^.

Multiple studies using various muscle strength indices have linked HGS with depressive symptoms among older adults. A cross-sectional study using data from the World Health Organization’s study on Global Ageing and Adult Health showed that dynapenia was associated with depression risk in middle-aged and elderly adults from six low-and middle-income countries^15^. Among Asian populations, HGS has been significantly associated with depressive symptoms. A Japanese study found that HGS was negatively associated with the Geriatric Depression Scale, although muscle mass was not associated with depression^16^. A Korean study found that the lowest HGS tertile was more associated with depressive symptoms than was the highest tertile^17^. Finally, a Chinese study reported that AWGS-based sarcopenia was associated with depressive symptoms^18^.

Our research corroborated these previous results, adding evidence that low HGS is correlated with increased depressed mood among elderly men. Several mechanisms could explain the association between low HGS and depressed mood. First, chronic low-grade inflammation is a common basis of both sarcopenia and depression^25^. Second, HGS and depression are positively associated with white matter integrity^26^. Third, malnutrition, a risk factor for sarcopenia, is common in geriatric depression^27^. Finally, low HGS is an important component of physical frailty, which is associated with late-life depression^28^.

Multiple reports have found an association between HGS and perceived stress or suicidal ideation among older adults. For example, sarcopenic obesity was associated with perceived stress in a Korean study, while perceived stress was negatively correlated with HGS in older adults in another study^29^. This correlation does not necessarily mean that HGS causes stress, as stress-related hormones can induce atrophic gene expression and cause muscle atrophy^30^.

Suicidal ideation is a strong predictor of suicide. Older adults tend to express suicidal thoughts to their physicians less frequently than do younger adults and physicians are more likely to miss intervention opportunities, contributing to high suicide rates in late life^31^. Suicidal ideation can be ameliorated; the Prevention of Suicide in Primary Care Elderly: Collaborative Trial intervention reduced suicidal ideation over a two-year period, improving depression outcomes among elderly primary-care patients^32^. This outcome demonstrates the importance of depression screening coupled with suicide-prevention protocols among older adults in primary care settings.

As low HGS or sarcopenia causes functional limitations and restricts activity, the condition could eventually lead to perceived burdensomeness, depression, and suicidal ideation. Here, we used a large, representative sample to demonstrate that low HGS was linked to suicidal ideation in older Korean men. Our work here followed up and corroborated our previous findings, using data from the Hallym Aging Study (n = 302)^33^. We were able to address a major weakness of our previous study, namely the small, non-representative sample size (data from Chuncheon, a small Korean city).

It is unclear why the strong link between low HGS and poor mental health was limited to elderly men. Possible explanations include higher cortisol stress responses among men than women, leading to greater inflammation and associated loss of muscle mass and strength^34^. Men may also feel more pressured to have a larger, muscular body as a symbol of power and may be more affected by age-related loss of strength than were women. However, we note that previous studies examining Western populations found a correlation between sarcopenia or low HGS and depressive symptoms in older women but not men^35^. Overall, this variation suggests that although Asian and Western populations both exhibit sex differences in the link between muscle strength and depression, the direction of these differences are distinct in each population. Another possible explanation for our results lies in the observation that significantly more older men than women were currently smoking; smoking is related to increased risk of sarcopenia^36^.

The present study had several limitations. First, we were unable to demonstrate causality. Low muscle strength could be a risk factor for depression and suicide, or the result of sedentary behaviour due to poor mental health. Therefore, we require prospective studies to elucidate mechanisms underlying the interaction of HGS and mental health. Second, the limitations of our data meant that we could not investigate potential associations between mental health and other components of sarcopenia (muscle mass or physical performance). Third, because KNHANES does not include a cognitive assessment questionnaire, we could not consider cognitive function as a confounding factor. However, KNHANES is a survey of relatively healthy community-dwelling individuals, and it does not include those in nursing homes and long-term care facilities. Therefore, it is very unlikely that participants with severe and moderate cognitive impairment would be included in our study. Finally, the 2015 and 2017 KNHANES (VI-3 and VII-2) did not include a standardised scale of depression and perceived stress. Instead, single item measures of depressive mood or perceived stress were used in the survey, which lead to a limitation of reliability for our study results. However, single item measures of depressive mood or perceived stress have been validated in previous studies^22,23^, and they have been widely used in survey research.

Nevertheless, our study also had important strengths. First, we addressed the issue of using different muscle strength indices, which could confound their relationship with health outcomes. Our use of three muscle strength indices (dynapenia, absolute HGS, and relative HGS) revealed consistent association with mental health indicators, suggesting reliable results. Second, our findings were based on a large, nationwide dataset that is representative of community-dwelling older adults in Korea, suggesting that the study is generalisable to the entire country.

In the present study, we successfully demonstrated that low HGS was associated with perceived stress among older adults of both sexes. Among men in particular, all muscle strength indices were correlated with perceived stress, depressed mood, and suicidal ideation. Overall, these results suggest that low HGS is a potential clinical marker of poor mental health in the elderly. The simplicity and cost-effectiveness of obtaining HGS make the measure a viable tool in primary care settings. Given our findings, we strongly recommend that physicians screen for psychiatric problems in older adults with low muscle strength.

## Data Availability

The KNHANES data can be downloaded from the website: https://knhanes.cdc.go.kr/knhanes/main.do.
